# Genotype–microbiome–metabolome associations in early childhood and their link to BMI

**DOI:** 10.1002/mlf2.12153

**Published:** 2024-12-24

**Authors:** Andrea Aparicio, Zheng Sun, Diane R. Gold, Jessica A. Lasky‐Su, Augusto A. Litonjua, Scott T. Weiss, Kathleen Lee‐Sarwar, Yang‐Yu Liu

**Affiliations:** ^1^ Department of Medicine, Brigham and Women's Hospital and Harvard Medical School Channing Division of Network Medicine Boston Massachusetts USA; ^2^ Department of Environmental Health Harvard T.H. Chan School of Public Health Boston Massachusetts USA; ^3^ Division of Pediatric Pulmonary Medicine, Golisano Children's Hospital at Strong University of Rochester Medical Center Rochester New York USA; ^4^ Division of Allergy and Clinical Immunology Brigham and Women's Hospital and Harvard Medical School Boston Massachusetts USA; ^5^ Vertex Pharmaceuticals Boston Massachusetts USA; ^6^ Center for Artificial Intelligence and Modeling, The Carl R. Woese Institute for Genomic Biology University of Illinois at Urbana‐Champaign Champaign Illinois USA

## Abstract

Through the analysis of data from children aged 6 months to 8 years enrolled in the Vitamin D Antenatal Asthma Reduction Trial (VDAART), significant simultaneous associations were identified between variants in the fragile histidine triad (*FHIT*) gene, children's body mass index, microbiome features related to obesity, and key lipids and amino acids. These patterns represent evidence of the genotype influence in shaping the host microbiome in developing stages and new potential biomarkers for childhood obesity, insulin resistance, and type 2 diabetes.

The human gut microbiome is thought to be acquired during birth^1^ and primarily shaped by environmental factors (e.g., diet) throughout the host's life^2^, ^3^. However, growing evidence obtained through microbiome–genome‐wide association studies (mGWAS) demonstrates the heritability of some microbiome features^4^, including taxa associated with body mass index (BMI)^5^. However, the human genome and gut microbiome contain a vast number of variants and species, making statistical power a challenge in mGWAS; a strict significance threshold of *p* < $5\times 10^{-8}$ is widely used. Additionally, the high microbiome diversity among individuals results in only a small fraction of species being shared among people and across populations. Consequently, only some mGWAS associations survive multiple testing corrections and, with a few exceptions^6^, replication among independent studies is rare.

While multi‐omic studies often integrate metabolomics as a link between genotypes and phenotypes^7^, simultaneous examinations of genotype, microbiome, and metabolome remain limited. Admittedly, multi‐omic integration increases the data dimensionality, complicating the analysis and interpretability of the results. Network methods offer an intuitive approach to this issue through the graphic representation of omics interconnections, mimicking the wiring between systems in the body^8^.

Early life physiology can shape long‐term human health, potentially predisposing individuals to negative outcomes. Factors disrupting young children's gut microbiome are linked to asthma, allergies, autism and childhood obesity^9^, ^10^. Similarly, early‐life metabolomics could shed light on the mechanisms leading to childhood obesity, autism, and IBD^11^. Recent studies have simultaneously explored early‐life microbiome and metabolome, often focusing on inflammation, asthma, or related outcomes^12^, ^13^.

Here, we integrate a multi‐omic pairwise association network in a cohort of 676 children in the longitudinal Vitamin D Antenatal Asthma Reduction Trial (VDAART), a multi‐site randomized trial of vitamin D supplementation during pregnancy. In this cohort, genetic and demographic information (Table S1), along with anthropometric measurements and blood and fecal samples, have been collected at several time points over 10 years (Figure 1A), yielding genotype, 16S microbiome, metabolome, and BMI data. We performed the analysis in three phases: (1) BMI–genotype–microbiome axis building through mGWAS, genotype–phenotype correlation exploration, and microbial differential abundance analyses. In this phase, we validated known genotype associations with the gut microbiome, found evidence of the genotype influence in shaping the host microbiome at a developmental stage, and established BMI–genotype associations through childhood. (2) Integration of the children's metabolomics into the BMI–genotype–microbiome axis by linearly regressing the relationship between the axis' variables and the metabolite abundances. In this phase, we identified metabolites, along with their corresponding pathways, associated with genotypic differences, and changes in BMI and microbiome variables, separately. (3) Multi‐omic network integration that summarizes all the pairwise associations identified in phases 1 and 2. The obtained network revealed novel genotype–microbiome–metabolome patterns associated with changes in BMI.

**Figure 1:**
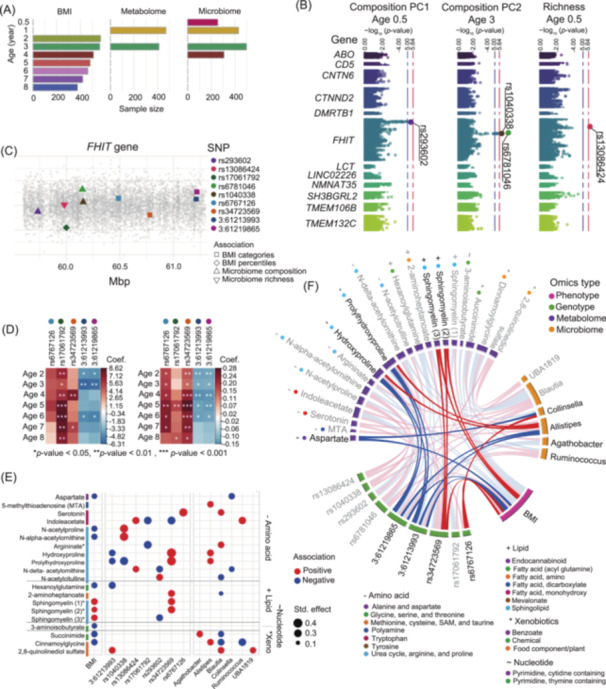
Genotype–Microbiome–Metabolome–BMI associations in early childhood. (A) Longitudinal data availability. Bars represent the number of available samples of each different data type, at every time point, between 0.5 and 8 years of age. (B) Four SNPs in the *FHIT* gene associated with microbiome features. At the suggestive level (*p* < 10^−5^), rs293602 is associated with microbiome composition PC1 at age 0.5. At the Bonferroni‐adjusted significant level (*p* < 2.29 × 10^−6^), rs6781046 and rs1040338 are associated with microbiome composition PC2 at age 3, and rs13086424 is associated with microbiome richness at age 0.5. (C) Locations of the SNPs associated with microbiome features and BMI measurements in the *FHIT* gene. Colors represent different SNPs, and shapes represent the associated variable. A relatively small region around 60 Mbp includes SNPs associated with microbiome features and BMI measurements. (D) The minor allele count (MAC) in five SNPs in the *FHIT* gene significantly and persistently (*p* < 0.01 for more than 5 consecutive measurements) associated with BMI measurements. BMI percentiles are positively associated with rs17061792 (left). BMI categories are positively associated with rs6767126 and rs34723569 and negatively associated with 3:61213993/3:61219865 (right). (E) 20 metabolites in 10 pathways simultaneously associated with at least two variables of different omics (BMI measurements, genotype, or microbiome features).** represents *p* < 0.01. (F) Network representation of all the pairwise associations found. Nodes are genetic, metabolome, microbiome, or BMI variables; links represent significant associations. We found two three‐node interconnections and one four‐node interconnection with coherent directional association, that is, an increase in one variable corresponds adequately to changes in others: (1) SNP rs34723569, lipid sphingomyelin, and BMI; (2) SNPs 3:61213993/3:61219865, amino acids hydroxyproline and prolyhydroxyproline, and genus *Allistipes*; and (3) SNP rs6767126, genus *Collinsella*, BMI, and aspartate aminotransferase.

The results obtained in each of the phases are detailed below. Notably, we found that a homozygous minor genotype in rs34723569 single‐nucleotide polymorphism (SNP) of the fragile histidine triad (*FHIT*) gene is associated with the enrichment of sphingomyelin (insulin inhibitor) and increased BMI. All the association analyses were adjusted for a priori selected potential covariates: participants' race, sex, and study site. For more details on the cohort, the analysis pipeline, and the statistical and informatics methods used, see the Supporting Information section.

In phase 1, to address the high‐dimensionality of the genotype data, we first identified 12 candidate genes previously found to be associated with microbiome features in independent studies^6^. Then, through a targeted microbiome–genome association study between all SNPs in the candidate genes and the alpha diversity and microbiome composition PCs (MCPCs) of microbiome samples at ages 0.5, 1, 3, and 4 years, we found three significant associations (*p* < ${2.29\times 10}^{-6}$, Bonferroni‐corrected) and one highly suggestive (*p* < $10^{-5}$) association (Figure 1B). All the associated variants are within the *FHIT* gene in chromosome 3, known to be associated with obesity^14^: (1) rs293602 is associated with MCPC1 at age 0.5; (2) rs6781046 and rs1040338–with highly correlated minor allele count (MAC) (Figure S1)—are associated with MCPC2 at age 3; and (3) rs13086424 is associated with microbiome richness at age 0.5. These “SNPs of interest” are located around the region 60 Mbp (Figure 1C). Notably, the strongest of these associations is (2), and MCPC2 is most heavily loaded with the genus *Blautia*, previously found to influence type 2 diabetes (T2D)^15^ and associated with visceral fat^16^.

Next, we looked for persistent associations (*p* < 0.01 for at least 5 consecutive measurements) between the MAC of all variants in the *FHIT* gene and BMI measurements throughout childhood. We found that rs17061792 is positively associated with BMI percentiles at ages 2 through 8; rs6767126 and rs34723569 are positively associated with BMI categories at ages 2 through 7, and 2 through 8, respectively; and 3:61213993 and 3:61219865 (with highly correlated MACs) are negatively associated with BMI percentiles at ages 2 through 6 (Figure 1C,D). We added these SNPs to the set of SNPs of interest (Figure 1C). Then, through independent MaAsLin analyses at the genus level, for every age available, we found six genera (which we call in the following the “genera of interest”) that are differentially abundant (*q* < 0.05) in relation to six of the SNPs of interest (Figure S2). At age 0.5 years, *UBA1819* is positively associated with rs13086424. At age 3 years, *Collinsella* and *Blautia* are positively associated with rs6767126 and with the pair rs6781046/rs104033, respectively. Also at age 3 years, *Allistipes* and *Agathobacter* are negatively associated with the pair 3:61213993/3:61219865. At age 4 years, *Ruminoccocus gnavus* group is positively associated with 3:61213993/3:61219865. Importantly, *R. gnavus* has also been previously associated with visceral fat^16^. Additionally, we looked for differential abundant taxa in relation to the BMI measurements but, consistent with previous results on this cohort^17^ we found no significant associations surviving multiple testing correction (Table S9).

In phase 2, we first fitted covariate‐adjusted linear models between the relative abundance of children's plasma metabolites and BMI at age 3, and identified a total of 25 metabolites in 21 metabolic “pathways of interest” (9 amino acids, 2 carbohydrates, 9 lipids, 2 nucleotides, and 3 xenobiotics) significantly associated (*p* < 0.01) with a BMI measurement (Figure S3). Then, the abundance of all metabolites in these 21 pathways of interest was separately tested for pairwise associations with BMI, the genera of interest at age 3 years, and the SNPs of interest. We found 11 amino acids, 5 lipids, 1 nucleotide, and 3 xenobiotics significantly associated (*p* < 0.01) with at least 2 variables of different omics, simultaneously (Figure 1E): 10 metabolites associated with genotype and microbiome variables; 7 associated with BMI and genotype; and 3 associated with BMI and microbiome.

Finally, in phase 3, we built the co‐association network in Figure 1F, where nodes represent metabolites, SNPs, genera, or BMI measurements and links represent the pairwise associations found in phases 1 and 2. We define a “loop” in the network as a closed connection between three or four nodes of different omics types. We deem a loop meaningful if the direction of the associations (or links) is coherent, that is, an increase in one variable corresponds adequately to changes in others. For example, a three‐node loop with two negative links and one positive link would be a meaningful loop. In this network, there are a total of four meaningful loops.

First, a triple positive association between the MAC in SNP rs34723569, the abundance of sphingomyelin, and BMI (Figure S4A). Sphingomyelin inhibits insulin action in humans^18^, which suggests that having a homozygous minor genotype at the rs34723569 locus might represent a higher risk of obesity and insulin resistance.

The second and third meaningful loops comprise the negative association of the MAC in SNPs 3:61213993/3:61219865 with hydroxyproline and prolyhydroxyproline (two amino acids in the urea cycle), and with the abundance of the genus *Allistipes*; *Allistipes* is positively associated with both amino acids (Figure S4C). Hydroxyproline is a dipeptidyl peptidase (DPPIV) inhibitor that is commonly used to treat T2D^19^, and the strain *Allistipes putredinis* has been found to be depleted in patients with obesity^20^. This suggests that a minor genotype at the 3:61213993/3:61219865 locus might represent a higher risk of obesity and insulin resistance. However, more in‐depth studies are needed to establish precise conclusions with respect to the microbiome part of the axis.

The fourth meaningful loop includes the positive association of the MAC in SNP rs6767126 with *Collinsella* and BMI, and the negative association of the two latter with amino acid aspartate (Figure S4B). *Collinsella* is a known proinflammatory genus whose enrichment is associated with T2D^15^; more research is necessary to determine the role of aspartate in our study's context.

To cross‐validate our findings, we used 70% of the available samples at age 3 years to linearly model the relationships between the variables identified in the four congruent loops: BMI; genera *Allistipes* and *Collinsella*; amino acids hydroxyproline, prolyhydroxyproline, aspartate; lipids sphingomyelin; and SNPs rs34723569, 3:61213993/3:61219865, and rs6767126. We fitted a total of 12 models; each one used one of the variables as the response and the rest as predictors (removing highly correlated variables of the same omic, for example, SNP 3:61213993 was not used as a predictor for 3:61219865 and vice versa); and all the models were adjusted for race, sex, and study site. Then, we generated predictions on the remaining 30% of the samples for each trained model and calculated the mean squared error (MSE) with respect to real measurements of the samples with complete data (*n* = 128). We found that all the models predicting microbiome or metabolome variables had an MSE < 1.5%, except for asparate and *Collinsella* (MSE of 2.4% and 4.4%, respectively, Figure S5); the models predicting genotype variables had the highest MSE, ranging from 3.5% to 11.7%. Because the network in Figure 1F was generated based on pairwise associations at multiple ages, the low MSE in the predictions at a single age point with considerable missingness (only 43% of the test set had complete measurements) confirms the robustness of our results.

In summary, we have presented confirming evidence that the genotype contributes to shaping the human microbiome even at developmental stages in young children. Furthermore, through a network integration of multi‐omic associations, we found three genotype–metabolome–microbiome patterns revealing potential markers and mechanisms for health outcomes related to child obesity and related complications such as insulin resistance and T2D. The described multi‐omics interconnections can be interpreted as redundancies that offer the possibility of more robust health screening, potentially facilitating early interventions design and ensuring healthy development of children.

To address the multiple testing burden of GWAS and multi‐omics studies, we adopted a sequential approach that progressively reduced the data dimensionality. Because the highest dimensionality resides in the children's genotype, we first selected a pool of 12 genes known to be associated with microbiome features in the mGWAS literature, allowing to relax the classical GWAS significance while maintaining a strict Bonferroni correction. Our mGWAS aimed to test the validity of existing results in the developing infant and early childhood microbiome rather than find new associations. We reduced the microbiome dimensionality through alpha diversity measurements and robust Aitchison PCA, while the metabolome dimensionality was reduced by preselecting metabolic pathways associated with the variables of interest and performing downstream analyses on individual metabolites pertaining to those pathways only.

Limitations of this study include the reduced statistical power for classical GWAS due to the relatively small sample size. Also, the lack of metagenome sequencing data on the stool samples limits the study to the genus level, obscuring potential differences in the abundance of individual species and their functionality. Finally, having the multiple omics measured at disparate time points, and a considerable missingness of data, hinders the possibility of performing a longitudinal analysis.

## AUTHOR CONTRIBUTIONS

**Andrea Aparicio**: Conceptualization (equal); formal analysis (equal); investigation (equal); methodology (equal); software (equal); writing—original draft (equal); and writing—review and editing (equal). **Zheng Sun**: Data curation (equal) and writing—review and editing (equal). **Diane R. Gold**: Data curation (equal) and writing—review and editing (equal). **Jessica A. Lasky‐Su**: Data curation (equal) and writing—review and editing (equal). **Augusto A. Litonjua**: Data curation (equal) and writing—review and editing (equal). **Scott T. Weiss**: Conceptualization (equal) and writing—review and editing (equal). **Kathleen Lee‐Sarwar**: Conceptualization (equal); methodology (equal); supervision (equal); and writing—review and editing (equal). **Yang‐Yu Liu**: Conceptualization (equal); methodology (equal); supervision (equal); and writing—review and editing (equal).

## ETHICS STATEMENT

The VDAART study protocol was approved by the institutional review boards at each participating institution and at Brigham and Women's Hospital. The study was conducted in accordance with the Declaration of Helsinki. Informed consent was obtained from all subjects involved in the study.

## CONFLICT OF INTERESTS

Andrea Aparicio, Zheng Sun, and Yang‐Yu Liu declare no conflicts of interest pertaining to this work. Diane R. Gold receives salary support from the NIH. Jessica A. Lasky‐Su is a scientific advisor to Precion Inc., TruDiagnostic Inc., and Ahara Corp. Scott T. Weiss receives royalties from UpToDate, Inc. and serves on the Board of Histolix, Inc.; Augusto A. Litonjua has received royalties from UpToDate, Inc.; and Kathleen Lee‐Sarwar is employed by and owns stock in Vertex Pharmaceuticals.

## Supporting information

Supporting information.

Supporting information.

## Data Availability

The multi‐omics data from VDAART are part of the Environmental influences on Child Health Outcomes (ECHO) consortium and ECHO consortium members and other interested scientists can obtain the data directly from the ECHO Data Coordinating Center. The processed data and/or code used in the data analysis conducted in this study are available from the corresponding author on reasonable request.
