# Supplementary material for: Genotype–microbiome–metabolome associations in early childhood and their link to BMI
Source: mLife. 2024 Dec 24;3(4):573–7. doi: 10.1002/mlf2.12153 (PMC11685832; doi:10.1002/mlf2.12153)
Supplement: Supplementary file 1 — Supporting information. [file MLF2-3-573-s002.docx]

# Supplemental Figures and tables

|  |  |  |  | BMI percentiles | | | | | | | Genetic principal components | | | | |
| --- | --- | --- | --- | --- | --- | --- | --- | --- | --- | --- | --- | --- | --- | --- | --- |
|  |  | N | % of cohort | Y2 | Y3 | Y4 | Y5 | Y6 | Y7 | Y8 | PC1 | PC2 | PC3 | PC4 | PC5 |
| Sex | Female | 310 | 47.7 | . | ns | ns | * | * | * | ns | ns | ns | ns | ns | ns |
|  | Male | 340 | 52.3 |  |  |  |  |  |  |  |  |  |  |  |  |
| Race/ Ethnicity | Hispanic/Latino or Other^+^ | 261 | 40.2 | *** | ns | * | . | ns | ns | . | *** | *** | * | * | ns |
|  | Black | 250 | 38.5 |  |  |  |  |  |  |  |  |  |  |  |  |
|  | White | 139 | 21.4 |  |  |  |  |  |  |  |  |  |  |  |  |
| Study Site | San Diego | 231 | 35.5 | *** | ns | ns | * | * | ns | . | *** | *** | *** | ** | . |
|  | Boston | 169 | 26 |  |  |  |  |  |  |  |  |  |  |  |  |
|  | St. Louis | 250 | 38.5 |  |  |  |  |  |  |  |  |  |  |  |  |

***: p-value < 0.001; **: p-value < 0.01; *: p-value < 0.05; . : p-value < 0.1.
^+^Race group “other” includes Hispanic and Latino children and children of races other than White, Black, or African American

**Table S1** Summary of children’s characteristics and associations with BMI measurements


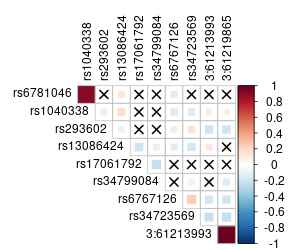


**Figure S1** Minor allele count correlation among the SNPs of interest. Black crosses denote non-significant correlations (p-value>0.01)


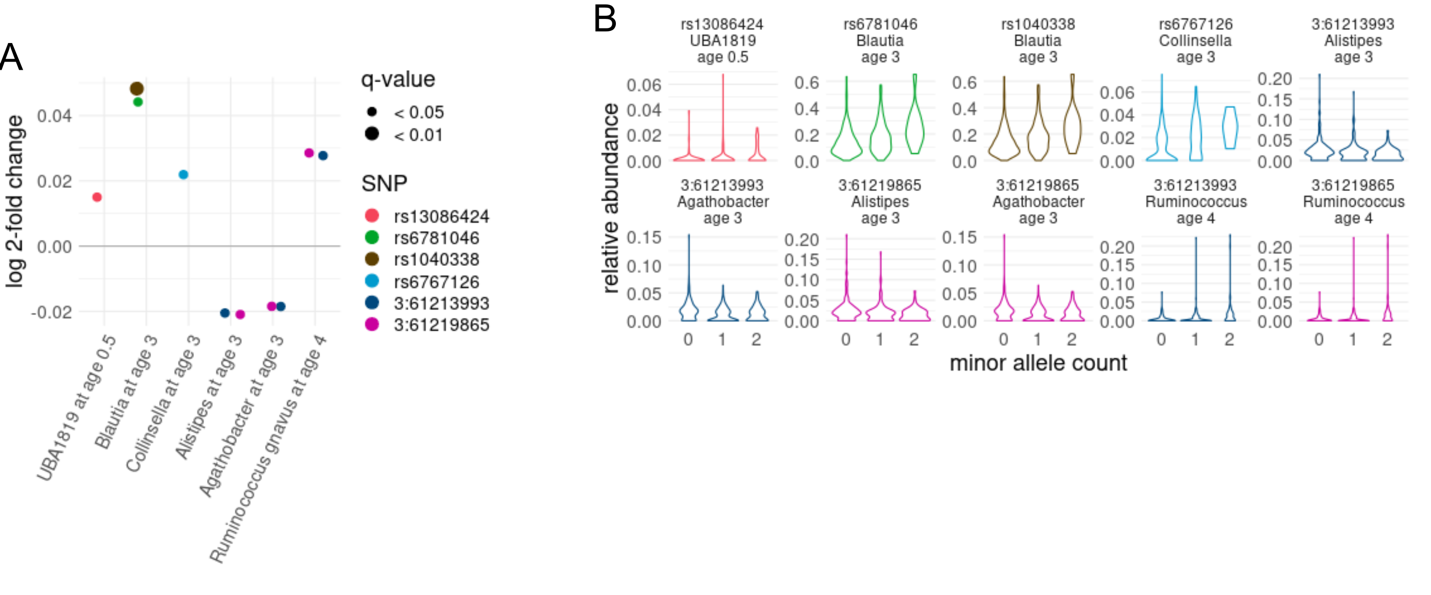


**Figure S2** Six microbiome genera at ages 0.5, 3, and 4 are differentially abundant with respect to the minor allele count (MAC) of the selected SNPs**.** Significance level is at q-val < 0.05, and MAC (0,1,2) is treated as a continuous variable. **A.** The strongest associations is between rs6781046 and rs1040338 and *Blautia* at age 3, the first having the also the strongest statistical significance, followed by 3:61213993/3:61219865 and *Ruminococcus gnavus* *group* at age 4. These associations have a positive coefficient. The rest of the associations are between *UBA1819* at age 0.5 and rs13086424; *Collinsella* at age 3 and rs6767126; and *Allistipes* and *Agathobacter* at age 3 and 3:61213993/3:61219865. The first two have positive coefficients while the latter two have negative coefficients. **B.** The relationship between the relative abundance of the differentially abundant bacteria and the MAC of the corresponding SNPs follows the coefficient sign pattern showed in panel A. Colors in this panel correspond to the SNPs in panel A. Highly correlated pairs of SNPs (rs6781046/rs1040338, and 3:61213993/3:61219865) show almost identical patterns. All genera, except for *Blautia*, have an important number of zero values. However, only *UBA1819* at age 0.5, and *Collinsella* at age 3 have overall low relative abundance. Out of the 6 genera associated with genotype, *UBA1819* presents the least robust association.


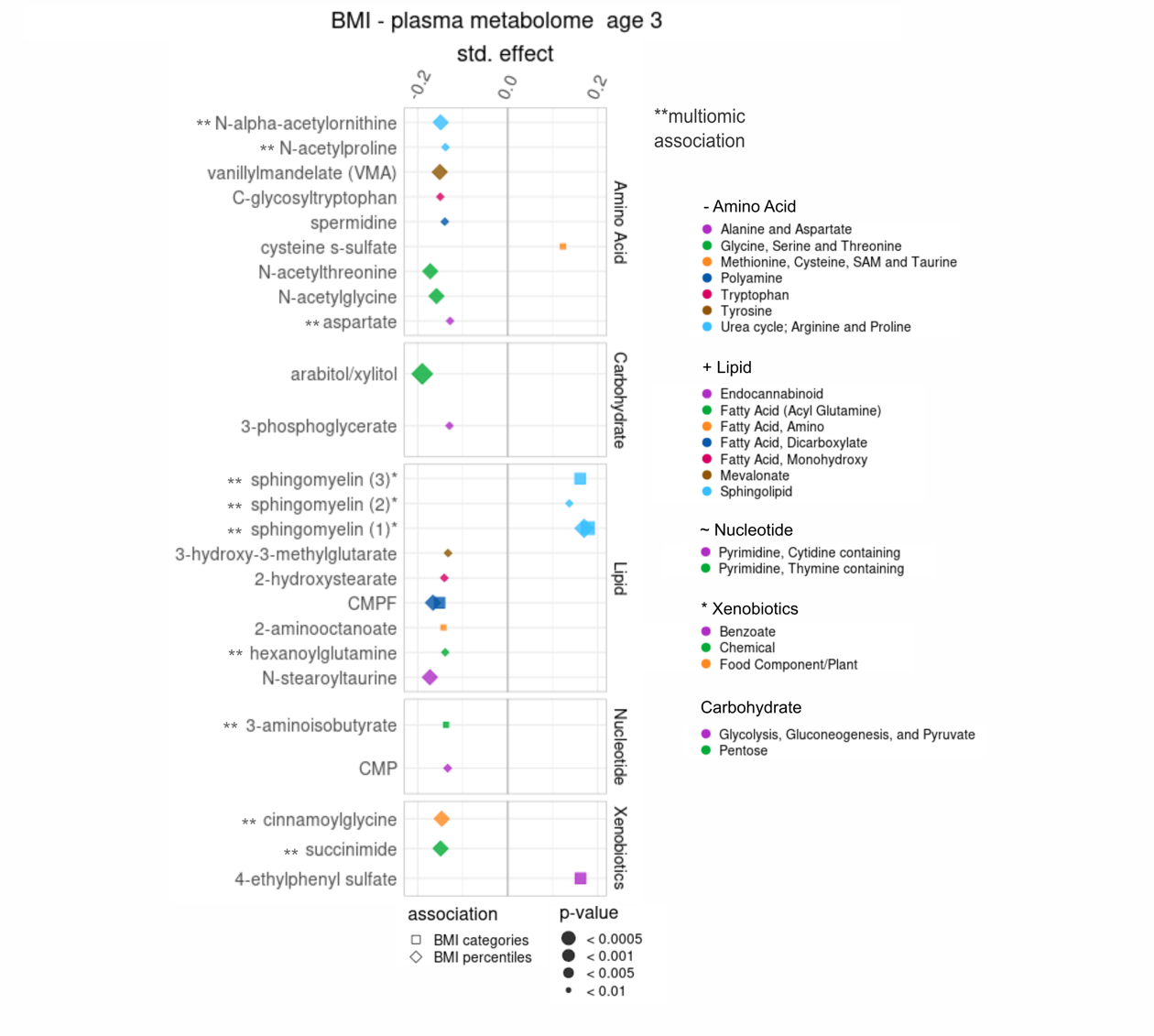


**Figure S3** Pathways in the plasma metabolome are simultaneously associated with BMI measurements. Associations with BMI percentiles are more prevalent than with BMI categories, but two lipids have significant associations with both. Amino acids and Lipids are the metabolite classes with most significant associations (n = 9, respectively). Cysteine s-sulfate in the Methione, Cysteine, SAM and Taurine metabolism pathway (Sulfur Amino Acids) is the only amino acid to have a positive association with BMI (categories), while all the other found associations are negative. The overall strongest association (negative) is between the carbohydrate arabitol/xylitol in the pentose pathway, and BMI percentiles, while the most consistent pathway association is between three lipids in the Sphingolipid pathway and both BMI measurements. In total, we identified 21 metabolic pathways, in 5 metabolite classes associated with BMI measurements. metabolism). Many of the metabolite associations involve more than one SNP per metabolite and at least 2 genera per metabolite.


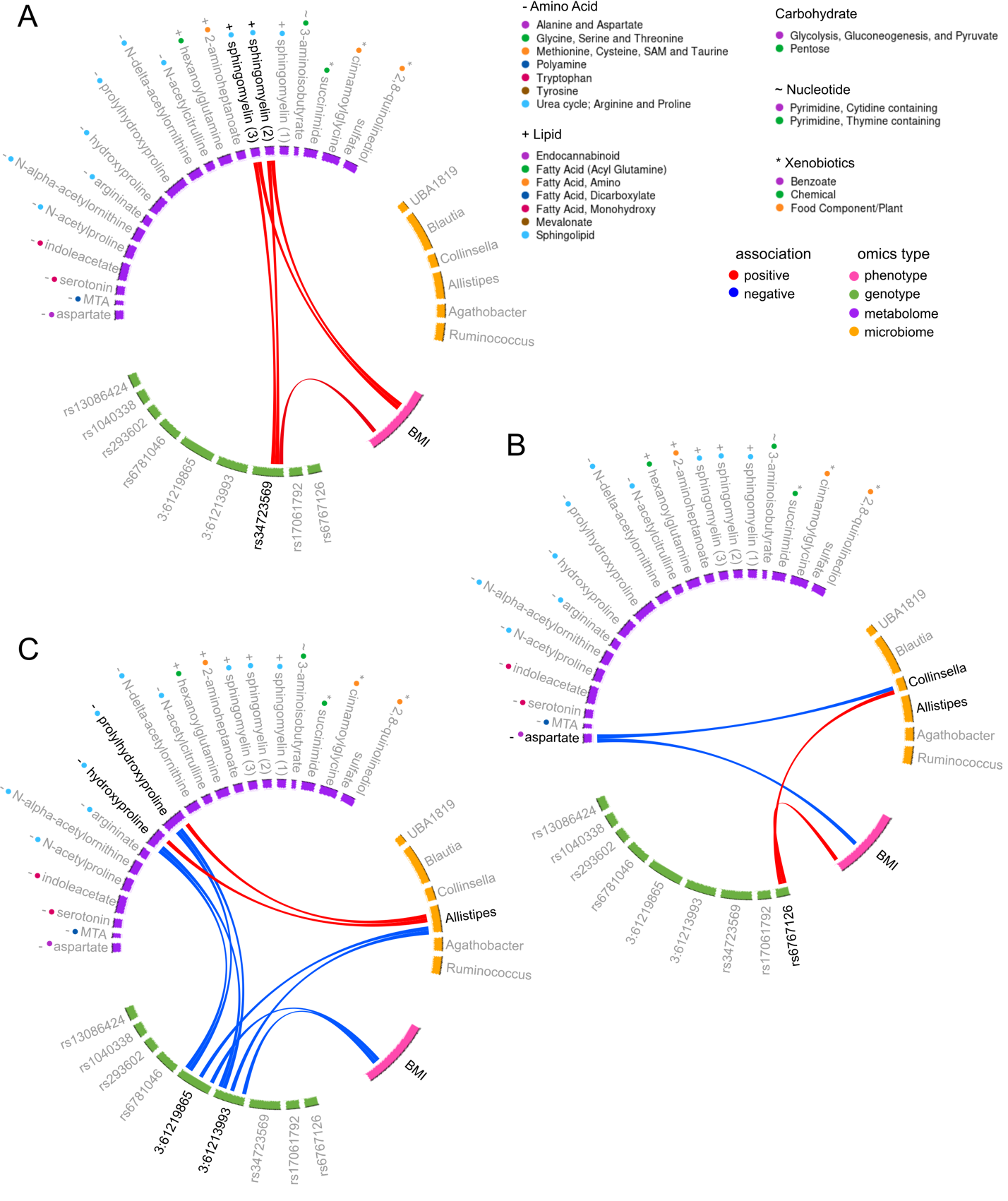


**Figure S4** Co-association network representation of the congruent associations found. Nodes are genetic, metabolome, microbiome or BMI variables, and links represent significant associations (red for positive and blue for negative).


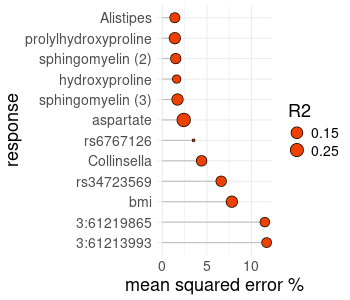


**Figure S5** Mean Squared Error and $R^{2}$ of predictions generated with linear models trained on 70% of the available data at age 3 years, on the remaining 30% of samples. Each model had one of the variables identified in the four meaningful loops as the response, and the rest as independent variables.

# Supplemental information

## Children’s characteristics

The children’s characteristics and BMI are summarized in**, Figure S6, Table S2, and Table S3.** Out of the 650 children with genotype data, 310 (47.7%) are females and 340 (52.3%) are males. The parent-reported race/ethnicity (**Methods 1**) is Hispanic, Latino or Other race for 261 children (40.2%), Black for 250 (38.5%), and White for 139 (21.4%). 231 (35.5%) of the participants were enrolled at the San Diego study site, 169 (26%) at the Boston study site, and 250 (38.5%) at the St. Louis study site (**Table 1**). The mean BMI across the participants is 17 for age 2; 16.6 for age 3; 16.4 for age 4; 16.4 for age 5; 16.8 for age 6; 17.2 for age 7; and 18 for age 8. The median BMI percentile across the participants is 55.5 for age 2; 68.2 for age 3; 70.4 for age 4; 69.8 for age 5; 71.4 for age 6; 69.1 for age 7; and 72.1 for age 8 (**Table S2**)**.**

BMI percentiles are highly correlated across timepoints when comparing the values for all children (**Figure S7.A**) but BMI categories and percentiles have a large within child variation across different ages (**Figure S7.B**). We found significant associations (p-value < 0.05) between several time points (ages 5, 6, and 7) of BMI percentiles and the children’s sex. We found a weak association between the children’s BMI at age 2 and their sex. However, sex is not significantly associated with any of the children’s first five genotype principal components. Children’s race and ethnicity are significantly associated with their BMI at ages 2, and 4, and weakly associated at ages 5, and 8. On the other hand, race and ethnicity are significantly associated with the first four genotype principal components. The study site is associated with the children’s BMI at ages 1, 5, and 6, and weakly associated at age 8. Finally, the study site is significantly associated with the first four genotype principal components, and weakly associated with the fifth. Therefore, we adjusted our downstream analyses for race/ethnicity, sex, and study site.


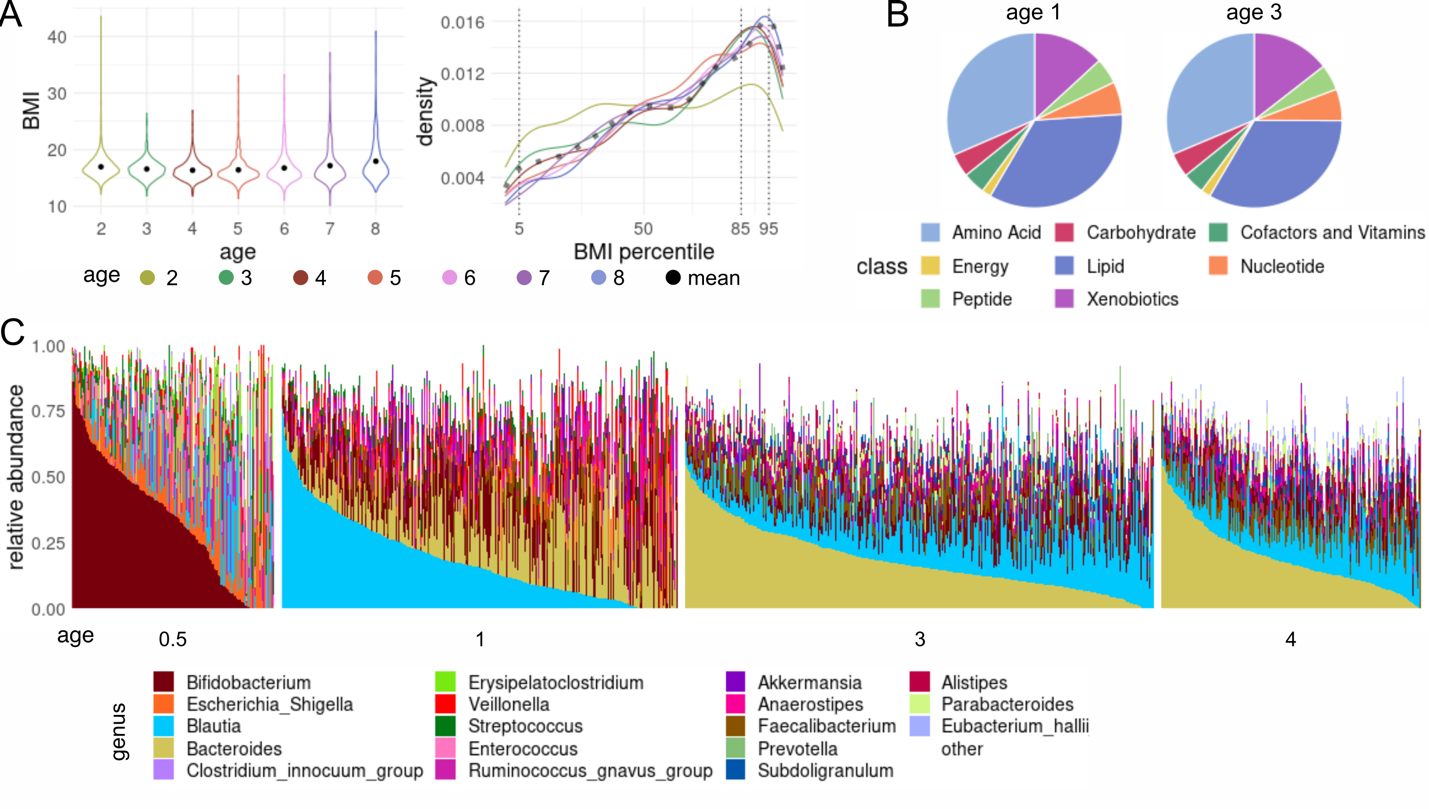


**Figure S6 A.** Distribution of child BMI per age (left), and of BMI percentiles per age (right). Black dots in the left panel represent the mean BMI per age, and black dotted line in the right panel represents the mean BMI percentile of all children at all ages available. Vertical thin dotted lines on the right panel delimit the BMI categories regions: BMI under the 5th percentile is considered underweight; between the 5^th^ and 85^th^ percentiles is considered normal weight; between the 85^th^ and 95^th^ percentiles is considered over-weight; above the 95^th^ percentile is considered obesity. For all ages, the BMI is skewed showing a higher prevalence of overweight individuals. **B.** Distribution of metabolite classes. The metabolite composition is dominated by lipids and amino acids. Children’s metabolome tended to be relatively stable between the two timepoints with the difference that slightly more Xenobiotics were present at age 3 relative to age 1. **C.** Microbiome composition of children at different ages. The colors represent the 10 genera with highest mean relative abundance per age group across all children. Every vertical bar represents an available stool sample for the corresponding age. As children grew, their microbiomes tended to be richer, and the composition tended to be more even. The infants’ microbiome at age 0.5 tended to be dominated by the genus *Bifidobacterium*, and by *Blautia* at age 1. At age 3 and 4 the microbiome was dominated by *Bacteroides* and, was more similar than between any other two timepoints.


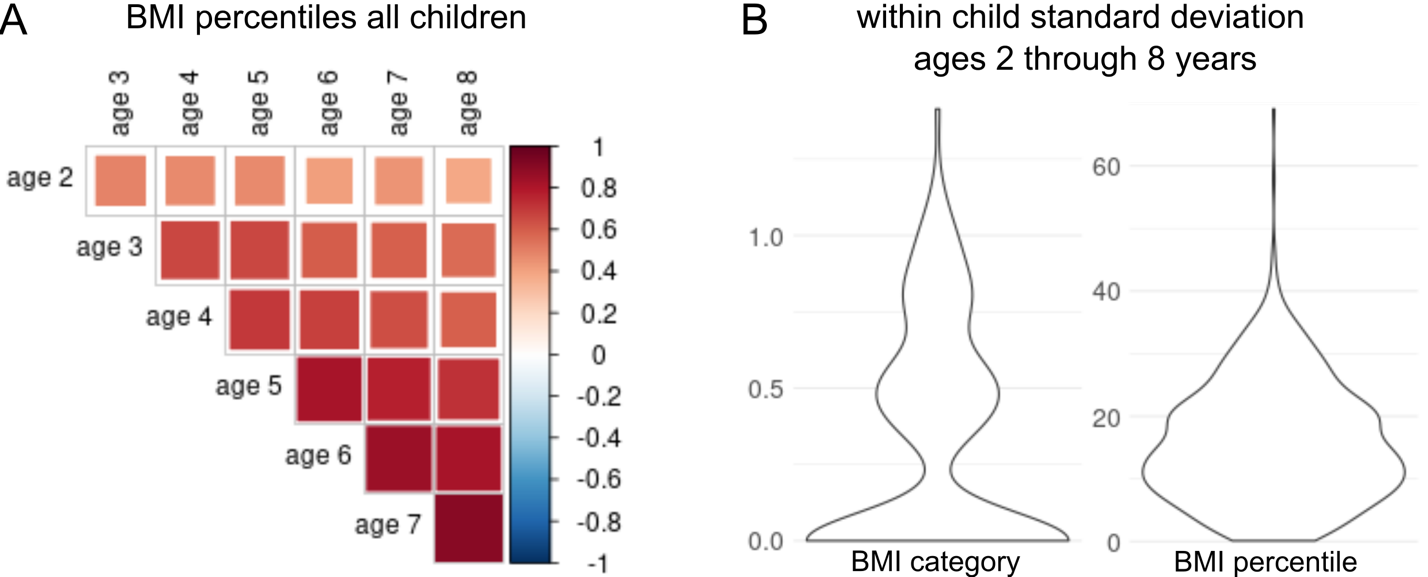


**Figure S7** BMI distribution in children from ages 2 through 8 years. **A.** Correlation of BMI percentiles per age. BMI percentiles across all children are highly correlated throughout the timepoints, especially towards older ages. **B.** Standard deviation of BMI categories and percentiles

## Variants in the *FHIT* gene are associated with microbiome features

To validate known genotype associations with the gut microbiome in our child cohort, we performed a targeted microbiome-genome association study between all SNPs in a pool of 12 candidate genes (**Methods 7**) and the alpha diversity and microbiome composition PCs (MCPCs) of microbiome samples at ages 0.5, 1, 3, and 4 years. Significance level was established at Bonferroni-corrected p-value < $2.29\times{10}^{-6}$, and a suggestive level at p-value < ${10}^{-5}$ (**Methods 9.2**). In total, three significant associations and one highly suggestive associations emerged, all within the fragile histidine triad diadenosine triphosphatase (*FHIT*) gene (**Figures 1.B**, **Figure S8**, **Figure S9**; **Table S4**): rs293602 is associated with MCPC1 at age 0.5 (p-value < ${4.5}^{-6}$); rs6781046 and rs1040338–with highly correlated minor allele count (MAC) and within 7430bp distance of each other (Figure S1**)**– are associated with MCPC2 at age 3 (p-value < ${2.2}^{-7}$and p-value < ${1.8}^{-6}$, respectively; and rs13086424 is associated with microbiome richness at age 0.5 (p-value < ${2.2}^{-6}$). The MCPCs associated with the SNPs of interest (see **Table** **S6** for SNP details) are most heavily loaded with the genus *Blautia* (**Figure S10)**.


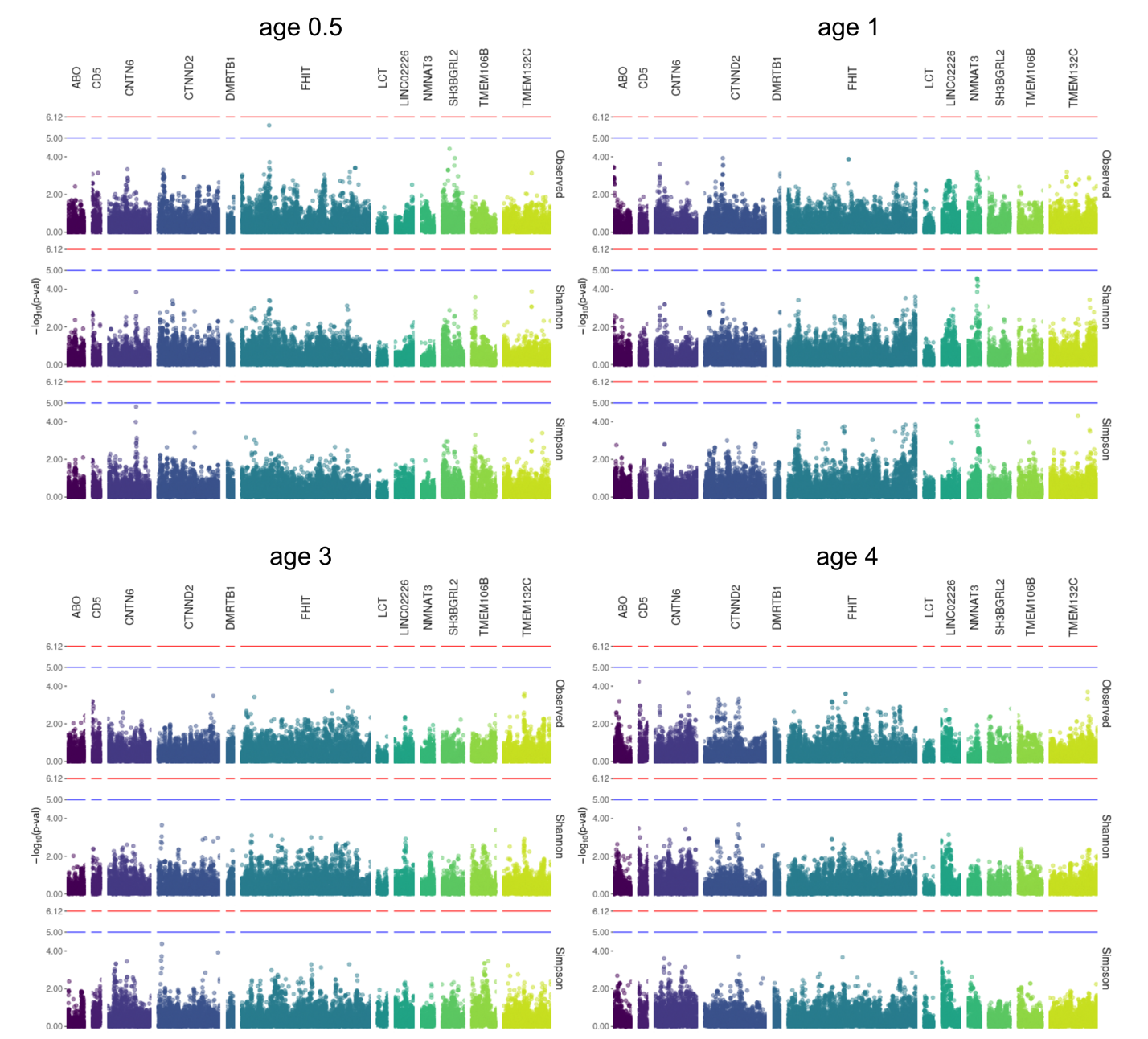


**Figure S8** microbiome alpha diversity GWAS results. Blue line denotes the suggestive level (p-value < ${10}^{-5}$), and the red line denotes the adjusted GWAS significant level (p-value < $2.29\times{10}^{-6}$). Only SNPs in the *FHIT* gene were above the suggestive level.


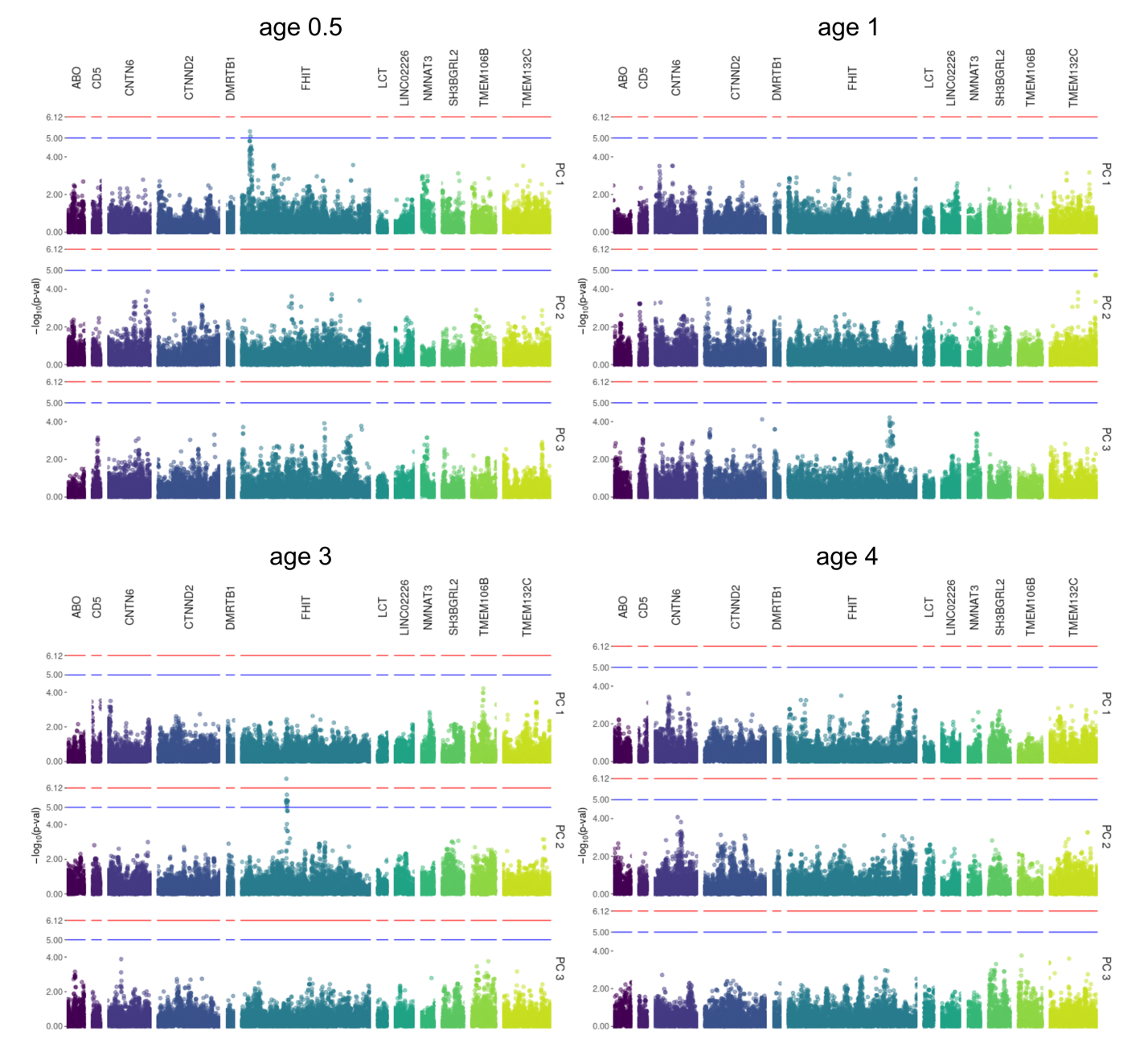


**Figure S9** microbiome composition principal components GWAS results. Blue line denotes the suggestive level (p-value < ${10}^{-5}$), and the red line denotes the adjusted GWAS significant level (p-value < $2.29\times{10}^{-6}$). Only SNPs in the *FHIT* gene were above the suggestive level.


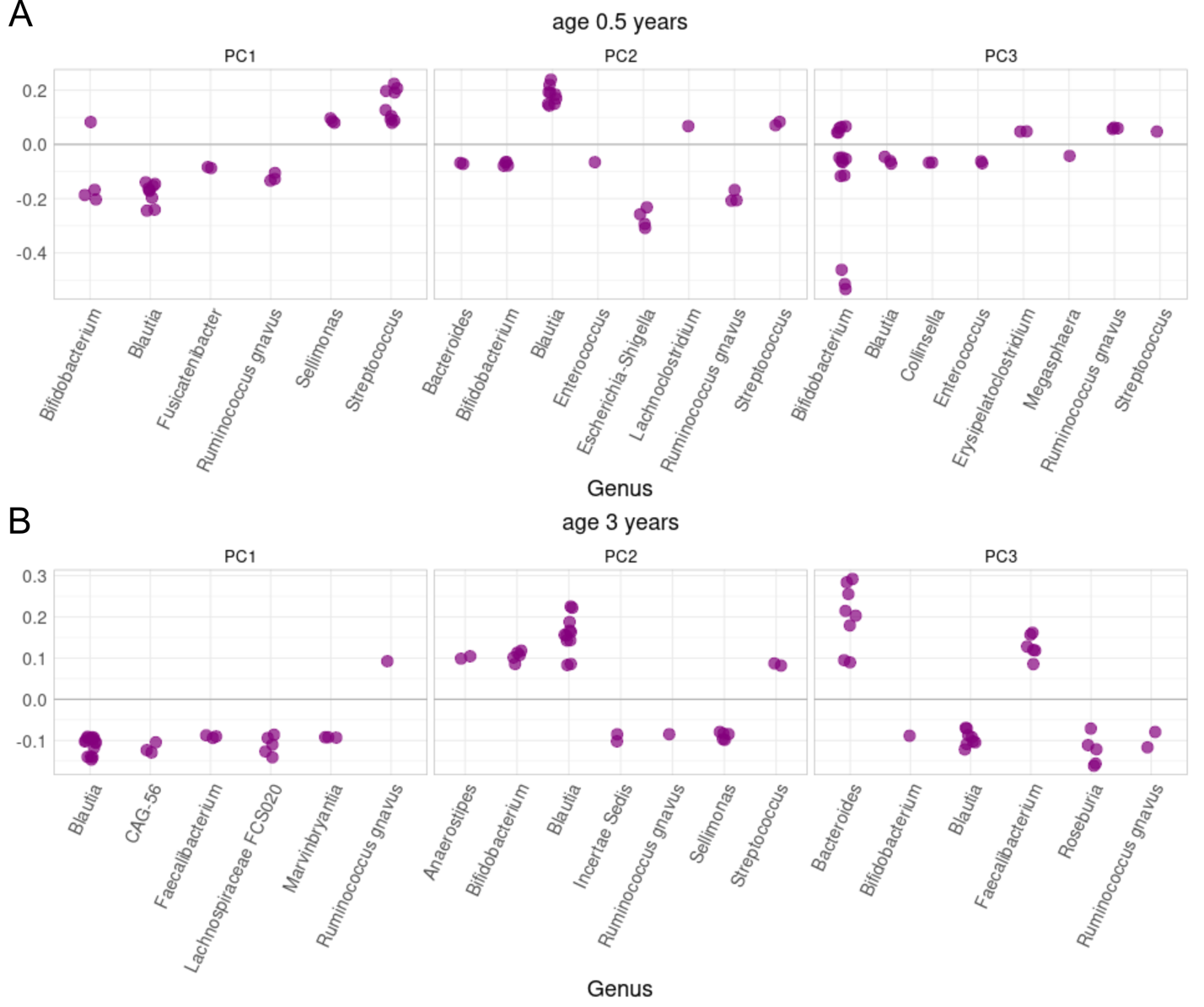


**Figure S10** Loadings of the microbiome composition principal components 1-3 at ages 0.5 (panel **a**) and 3 years (panel **b**). Principal components are obtained at the lowest taxonomic rank, points in the panels represent the 30 heaviest loadings (absolute value) of every PC at every age, grouped by the genus they belong to. PC1 at age 0.5, and PC3 at age 3, significantly associated with variants in the *FHIT* gene are most heavily loaded with members of the genus *Blautia*.

## Loci in the *FHIT* gene are associated with children´s BMI throughout childhood

We first pre-selected SNPs that we found were persistently correlated with BMI measurements (we call a correlation persistent when it is statistically significant–p-value < 0.01, for more than 5 consecutive time points of at least one of the measurements: BMI, BMI categories, or BMI percentiles, and the association maintains the same direction for all time points across both measurements). The frequency of allele T in rs17061792 is positively correlated with the children’s BMI percentiles; the frequency of allele C in rs6767126 and of allele A in rs34723569 are positively correlated with the children’s BMI and BMI categories, respectively; and the frequency of allele T and G in 3:61213993, and 3:61219865, respectively, is negatively correlated with BMI percentiles (**Figure S11** - **Figure S22**, and **Table S5** contain all SNPs with at least one significant association with a BMI measurement).

To confirm the associations, we ran covariate-adjusted linear regression on the minor allele count of the persistently correlated SNPs with respect to BMI percentiles and categories at every age, with a relaxed p-value < 0.05 (**Methods 9.3)**. All 4 SNPs correlated with BMI measurements were significantly associated with at least 5 consecutive timepoints of BMI measurement with a relaxed significance level of p-value < 0.05 (**Table S6**): The frequency of allele T in rs17061792 is positively associated with the children’s BMI percentiles (all p-values for ages 2 through 8 < 0.05); the frequency of allele C in rs6767126 and of allele A in rs34723569 are positively associated with the children’s BMI categories (p-values < 0.05 for ages 2 through 7, and 2 through 8 respectively); and the frequency of allele T and G in 3:61213993, and 3:61219865, respectively, is negatively associated with the BMI percentiles (p-values < 0.05 for ages 2 through 6). The minor allele counts of 3:61213993/3:61219865 are highly correlated (**Figure S1**).


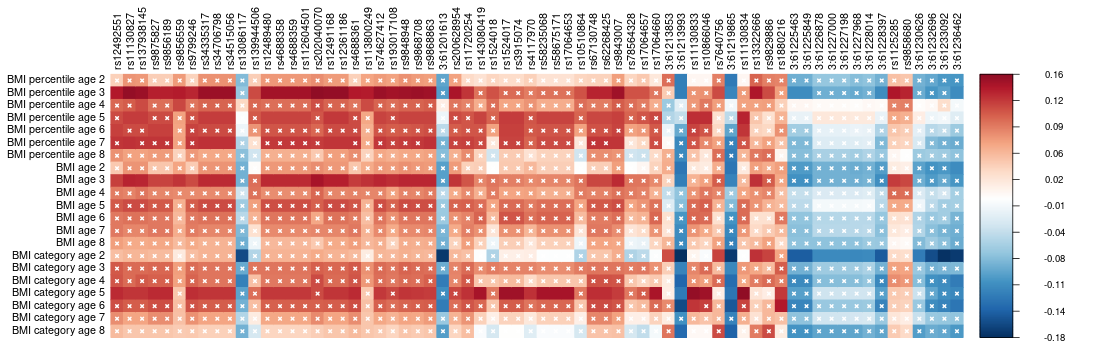


**Figure S11** SNPs in the *FHIT* gene with at least one significant correlation (p-val<0.01) with a BMI measurement. Colors in the heatmap represent the spearman correlation strength. White crosses denote non-significant correlations.


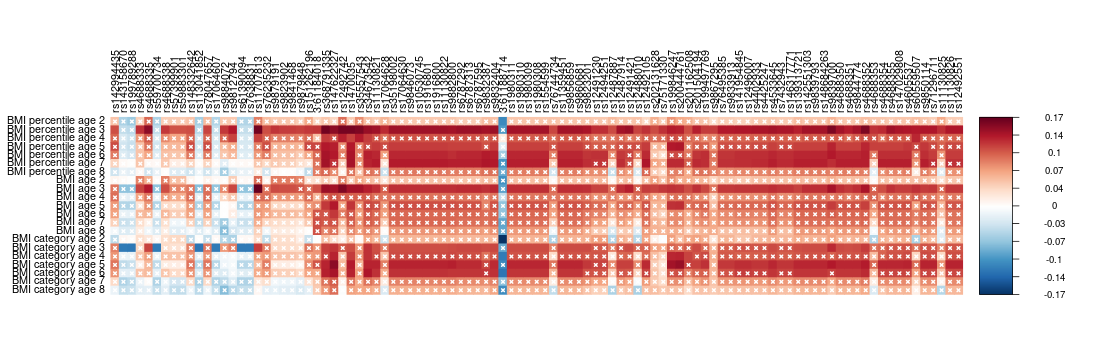


**Figure S12** SNPs in the *FHIT* gene with at least one significant correlation (p-val<0.01) with a BMI measurement. Colors in the heatmap represent the spearman correlation strength. White crosses denote non-significant correlations.


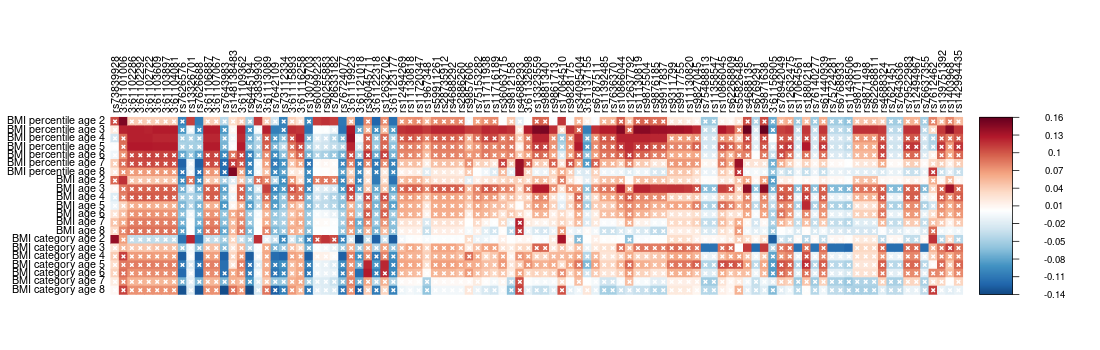


**Figure S13** SNPs in the *FHIT* gene with at least one significant correlation (p-val<0.01) with a BMI measurement. Colors in the heatmap represent the spearman correlation strength. White crosses denote non-significant correlations.


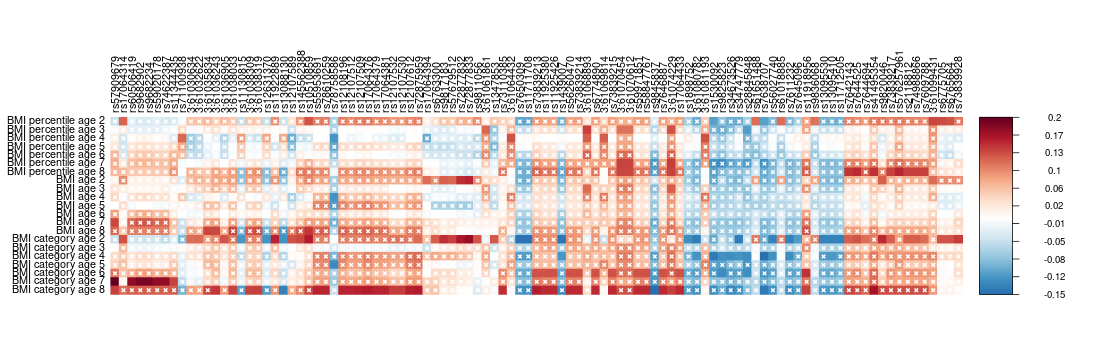


**Figure S14** SNPs in the *FHIT* gene with at least one significant correlation (p-val<0.01) with a BMI measurement. Colors in the heatmap represent the spearman correlation strength. White crosses denote non-significant correlations.


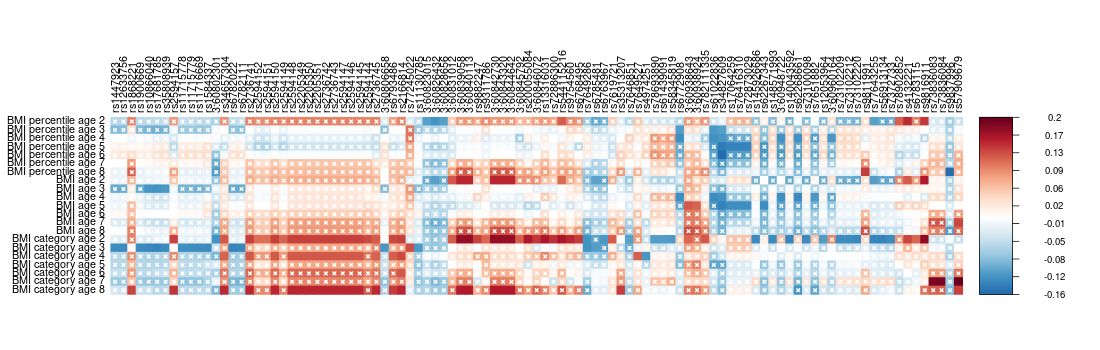


**Figure S15** SNPs in the *FHIT* gene with at least one significant correlation (p-val<0.01) with a BMI measurement. Colors in the heatmap represent the spearman correlation strength. White crosses denote non-significant correlations.


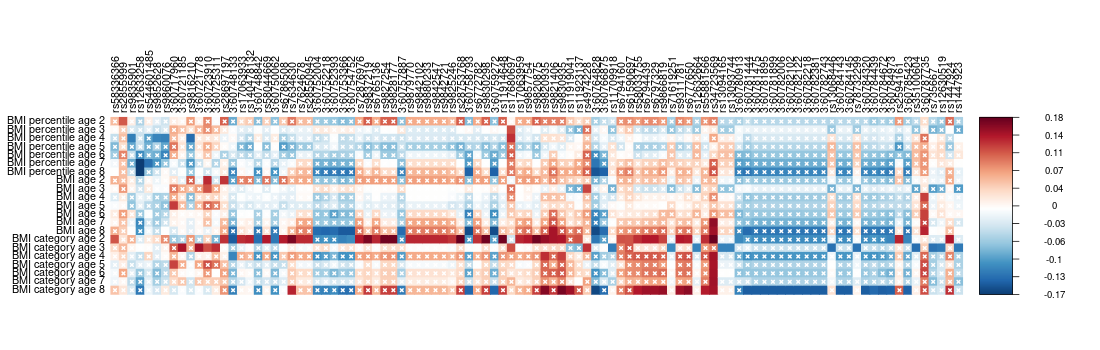


**Figure S16** SNPs in the *FHIT* gene with at least one significant correlation (p-val<0.01) with a BMI measurement. Colors in the heatmap represent the spearman correlation strength. White crosses denote non-significant correlations.


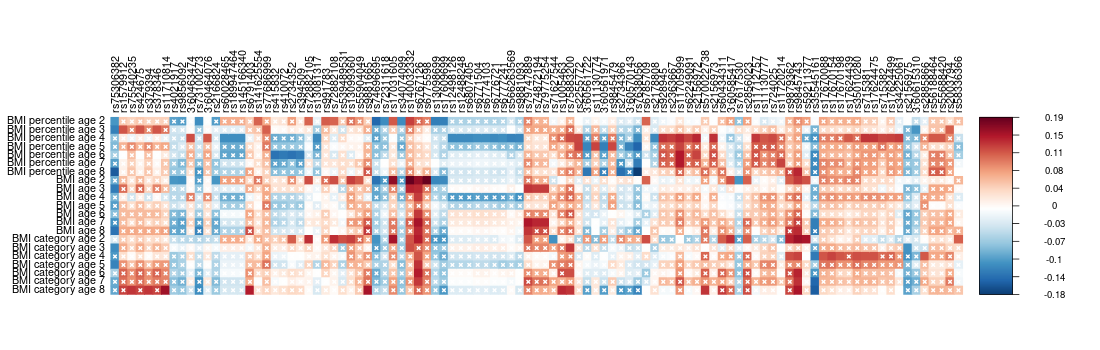


**Figure S17** SNPs in the *FHIT* gene with at least one significant correlation (p-val<0.01) with a BMI measurement. Colors in the heatmap represent the spearman correlation strength. White crosses denote non-significant correlations.


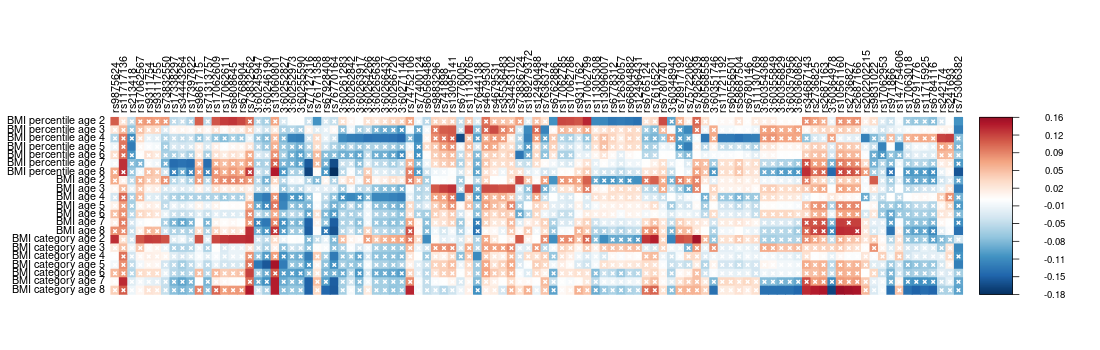


**Figure S18** SNPs in the *FHIT* gene with at least one significant correlation (p-val<0.01) with a BMI measurement. Colors in the heatmap represent the spearman correlation strength. White crosses denote non-significant correlations.


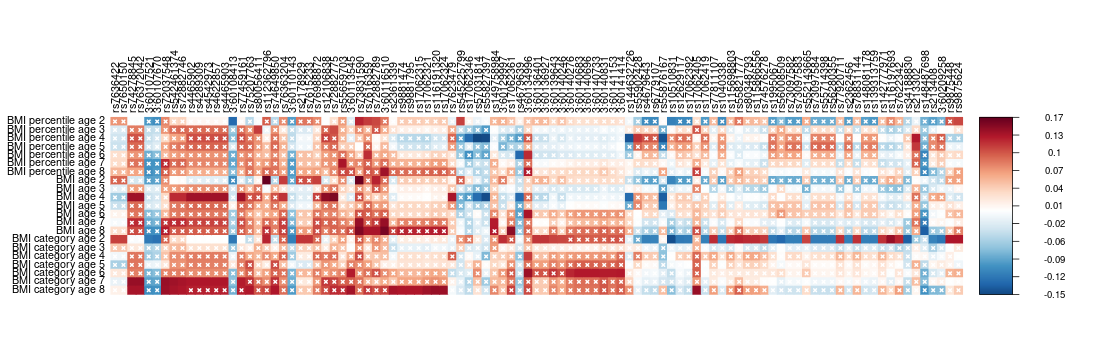


**Figure S19** SNPs in the *FHIT* gene with at least one significant correlation (p-val<0.01) with a BMI measurement. Colors in the heatmap represent the spearman correlation strength. White crosses denote non-significant correlations.


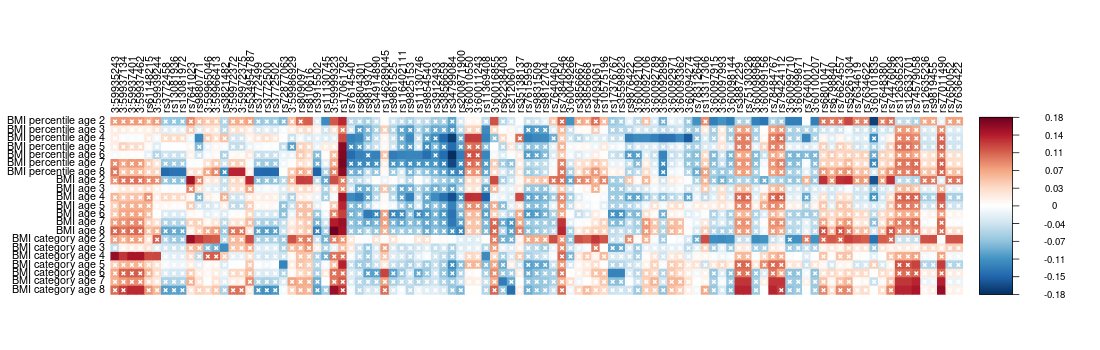


**Figure S20** SNPs in the *FHIT* gene with at least one significant correlation (p-val<0.01) with a BMI measurement. Colors in the heatmap represent the spearman correlation strength. White crosses denote non-significant correlations.


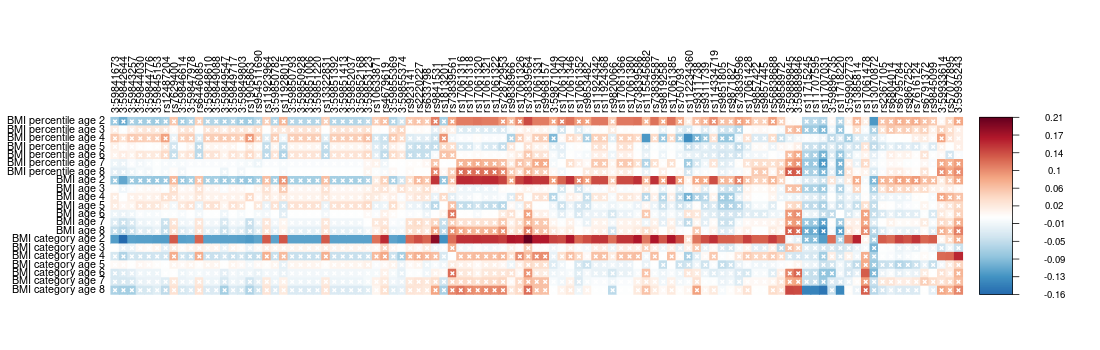


**Figure S21** SNPs in the *FHIT* gene with at least one significant correlation (p-val<0.01) with a BMI measurement. Colors in the heatmap represent the spearman correlation strength. White crosses denote non-significant correlations.


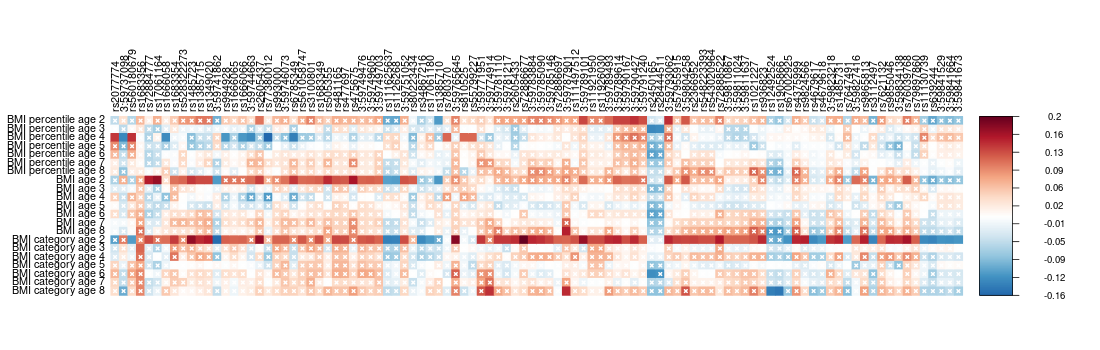


**Figure S22** SNPs in the *FHIT* gene with at least one significant correlation (p-val<0.01) with a BMI measurement. Colors in the heatmap represent the spearman correlation strength. White crosses denote non-significant correlations.

## Variants in the *FHIT* gene are associated with microbiome features

A positive association means that high MAC is associated with the genus’ enrichment. All genera of interest, except for *Blautia*, were absent in a large proportion of samples at the significantly associated ages (zero and mean abundances are summarized in **Table S9**). However, their relative abundance is substantial across the samples, except for *Collinsella* at age 3 and *UBA1819* at age 0.5.

## Preselection of metabolic pathways associated with BMI measurements.

To preselect metabolic pathways of interest, we fitted covariate adjusted linear models between children’s plasma metabolites relative abundance and BMI at age 3. We found a total of 25 plasma metabolites (9 amino acids, 2 carbohydrates, 9 lipids, 2 nucleotides and 3 xenobiotics) that were significantly associated (p-value < 0.01) with either BMI percentile, BMI category, or both (**Figure S3**; **Tables S8 and S11**). These 25 metabolites span 21 metabolic pathways that we carried forward in downstream analyses; we will refer to these pathways as pathways of interest in the following. Notably, two forms of Sphingolipid Sphingomyelin (1) [d18:1/20:2, d18:2/20:1, d16:1/22:2]; and (2) Sphingomyelin [d18:1/22:2, d18:2/22:1, d16:1/24:2]), and CMPF (a fatty acid in the dicarboxylate metabolism), are associated with both BMI measurements with similar significance. Most of the other associations are between plasma metabolite relative abundances and BMI percentiles; the two exceptions with the strongest associations are another form of sphingomyelin ([d18:2/14:0, d18:1/14:1]) and the xenobiotic benzoate 4-ethylphenyl sulfate, both of which are positively associated with BMI categories. The Xenobiotic 4-ethylphenyl sulfate, along with the amino acid Cysteine s-sulfate in the Methione, Cysteine, SAM and Taurine metabolism pathway (Sulfur Amino Acids) and the three forms of Sphingomyelin mentioned above are the only metabolites to have positive associations with BMI measurements; all the other observed associations are negative. While we observed few associations between carbohydrates and BMI measurements, arabytol/xylitol has the strongest overall association that we found. This carbohydrate is in the pentose pathway and is negatively associated with BMI percentiles. Sphingolipid metabolism is the pathway with the highest number of associations (n=3). All pairwise associations between metabolites abundances and the other omics types are shown in **Figure S23**.


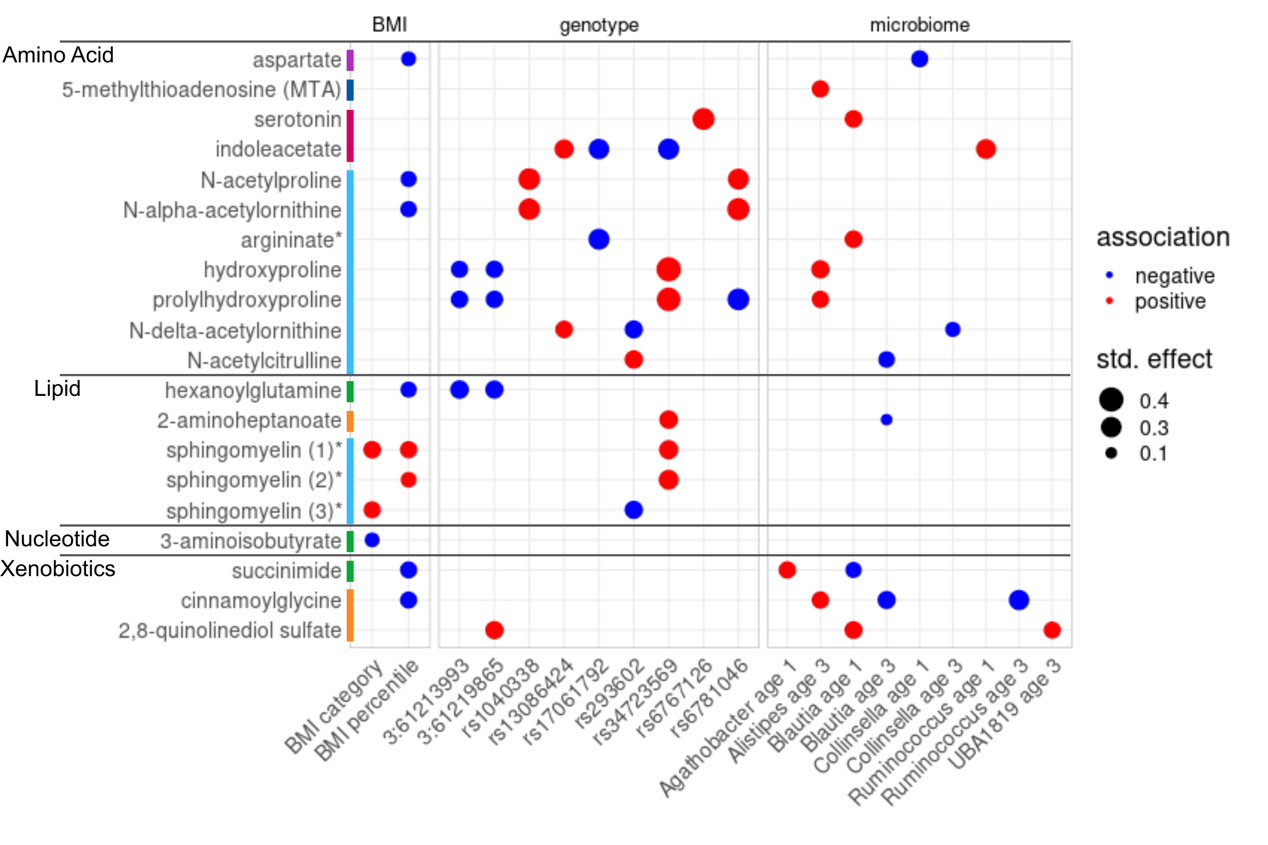


**Figure S23** Metabolites associated with at least one variable in two different types of omics data (BMI measurements, genotype, or microbiome). Sizes of the dots represent the standardized effect calculated using the coefficient β that resulted from fitting a linear model between the two corresponding variables as β*sd(explanatory variable measurement in all children)/sd(metabolite abundance in all children) to allow magnitude comparison (std. effect in the panels).

# Materials and methods

## VDAART and children’s characteristics

We analyzed data from the offspring of participants in the VDAART clinical trial (NCT00920621) [1]. VDAART is a randomized controlled trial of Vitamin D supplementation during pregnancy to prevent asthma in offspring conducted in three sites in the United States: Boston, MA; San Diego, CA; and St. Louis, MO., where stool and blood samples were collected. The study protocol was approved by the IRBs at each participating institution and all participants provided written informed consent [2]. VDAART enrolled women in the first trimester of pregnancy (n=876) in 2009. The available subjects’ characteristics from the initial enrollment questionnaire are the family household income, mother’s education level, and parents’ and children’s race and ethnicity. Race and ethnicity information was originally collected in VDAART because they are determinants of the circulating 25-hydroxyvitamin D levels; the race and ethnicity of parents was self-reported and that of every child participant was reported by their parent. Parents were asked to first categorize themselves and their child as either Hispanic or non-Hispanic, and then to categorize their race into prespecified categories. Race/ethnicity (called “race” hereafter) groups were collapsed into 3 groups for the analysis: Black or African American (called “Black” hereafter), White, non-Hispanic (called “White” hereafter), and Hispanic and children of other races (called “Other race” hereafter). After delivery, the offspring’s genotype, microbiome, metabolome, and body mass index data were collected from 6 months to 8 years old during annual clinic visits.

## BMI measurements

From age 2 through 8 years, the weight and height of children was recorded yearly. BMI was calculated as $BMI=weight (\mathrm{kg})/ {height (m)}^{2}$. BMI percentiles were calculated using the R package childsds [3] according to the children’s age and sex. “BMI categories” were obtained according to the BMI Weight Status Categories [4] as: underweight (BMI < 5^th^ percentile); normal weight (5^th^ percentile $\leq$ BMI < 85^th^ percentile); overweight (85^th^ percentile $\leq$ BMI < 95^th^ percentile); and obese (BMI $\geq$95^th^ percentile). We use the term “BMI measurements” to refer to either one of the BMI percentiles or categories generically.

## Metabolome profiling

Untargeted metabolomic profiling was performed at Metabolon (Research Triangle Park, NC) [5] on plasma collected at ages 1 and 3 years (n=470 and n=411, respectively) [6]. Results were expressed as relative abundance. Based on the assumption that missingness is due to low signal intensity, missing values were replaced with half of the minimum relative abundance observed for the metabolite in question [7]. Finally, relative abundances were log_10_ normalized and Pareto-scaled (mean-centered and divided by the square root of the standard deviation).

## Stool samples

At ages 0.5, 1, 3 and 4 years old, (n=256, n=436, n=506, n=314, respectively) child participants of VDAART provided a stool sample. Their parents were asked to collect a 0.5 teaspoon-sized sample 1 to 2 days before a study visit and store the sample in a home freezer before transport with a freezer pack to the study site. Stool was not collected if participants had used antibiotics in the past 7 days. After delivery to the study site, stool samples were immediately stored at -80°C. Microbiome profiling was performed by sequencing the 16S rRNA hypervariable region 4 (V4 515F/816R region) on the Illumina MiSeq platform at Partners Personalized Medicine (Boston, MA).

## Genotype principal components and minor allele count

Genotyping was performed in VDAART participants using the Illumina Infinium HumanOmniExpressExome Bead chip. Imputation was performed using Minimac [8] on the Michigan Imputation Server with the 1000 Genomes reference panel. Genotype principal components analysis (PCA) was performed using LASER, which analyzes sequence reads of each sample and places the sample into a reference PCA space constructed using genotypes of a set of reference individuals [9]. Minor allele count of SNPs was obtained using PLINK version 1.9.

## Microbiome composition and PCs

Microbiome samples were centered log-ratio transformed on only their non-zero values, and dimensionality reduction to three microbiome composition PCs (MCPCs) was performed through Robust Aitchison PCA [10] (function auto-rpca in Python package Gemelli).

## Candidate genes

Instead of performing an association study on the whole children’s genome, we focused on candidate genes identified in a comprehensive literature review  [11]. The selection criteria is that variants in every gene must have been associated with microbiome features in at least two independent previously published studies. This yielded pool of 12 candidate genes: *DMRTB1* in chromosome 1; *LCT* in chromosome 2; *CNTN6*, *FHIT*, and *NMNAT3* in chromosome 3; *LINC02226* and *CTNND2* in chromosome 5; *SH3BGRL2* in chromosome 6; *TMEM106B* in chromosome 7; *ABO* in chromosome 9; *CD5* in chromosome 11; and *TMEM132C* in chromosome 12. References and details in **Table S13**.

## Sequential knowledge-based data reduction

The preselection of variables started with the genotype, which has the highest dimensionality. The sequential steps are: 1. Select candidate genes with known associations with microbiome features; 2. Identify subsets of SNPs (of interest) significantly associated with microbiome richness and composition, and with children’s BMI 3. Identify sets of genera (of interest), differentially abundant with respect to the SNPs of interest’s MAC. 4. Look for metabolomic simultaneous associations with BMI, and genera and SNPs of interest.

## Statistical analysis

Unless specifically noted, we used a cut-off p-value of 0.05 to deem results statistically significant. All association analyses were adjusted for *a priori* selected potential covariates: participants’ race, sex, and study site.

### Alpha diversity

Alpha diversity measures are estimates of an individual’s taxonomic diversity. We computed the observed richness (number of different taxa present in each sample), Shannon, and Simpson indices (both incorporate the richness and evenness of samples) using the R Phyloseq package [12].

### Targeted microbiome genome-wide association study

Using PLINK’s association analysis functionality, we applied covariate-adjusted genome-wide association tests to the 21834 variants in the candidate genes, and the three PCs of microbiome composition at every available age, and alpha diversity measures. Accounting for the total number of variants in the candidate genes, a Bonferroni-corrected significance level was established at p-value < $2.29\times{10}^{-6}$, and a suggestive level at p-value < ${10}^{-5}$.

### SNP-BMI correlations

We calculated the Spearman correlation between the genotype of every SNP in the genes associated with microbiome features, and the children’s BMI, BMI percentile, and BMI category at every available age. Because we focused on significant associations from the mGWAS analysis with a more rigorous significance level, SNPs with a correlation p-value < 0.01 for more than five consecutive BMI, percentiles, or categories timepoints were selected. Persistent and significant associations are those that meet the significance criteria and preserve the association direction for all timepoints across all BMI measurements.

### Linear regression

Significant and persistent correlations between SNPs and BMI measurements were confirmed via covariate adjusted linear models (one for every available BMI measurement across time-points), using the lm R function, with a relaxed significance level of p-value < 0.05. We also used covariate-adjusted linear models to search for associations between the children’s metabolites and BMI, genotype, and genera relative abundance with p-value < 0.01.

### Differential abundance

To search for associations between the subjects’ genotype and BMI, and the abundance of specific taxa, we used MaAsLin2 and its corresponding R package, treating the minor allele frequency at a particular locus as a continuous variable. MaAsLin is a multivariate association method that uses additive linear models to detect associations between specific groups and the abundance of taxa, simultaneously treating all the present taxa as outcomes [13]. We used the false discovery rate (FDR) method to adjust the *p*-values for multiple comparisons. The association analyses were performed on genus-level data.

## Network integration

Based on the association analysis, we built a network using the R package *igraph* [14], where the weight of the edges (-1 or 1) represents the direction of the association, and every node is a tested variable. A loop is a connected component (a set of nodes where any of them can reach any other by traversing edges) with the minimal number of edges. A meaningful loop is one that has congruent edge weights, i.e., an increase or decrease in one variable will lead to a congruent change in the other variables, as represented by the direction of the associations. Examples of meaningful loops are a three-node loop with two negative and one positive associations, or a loop with all positive associations.

## Cross-validation

## To cross validate our results, we performed the following steps:

1. Obtain the data at age 3 years of the variables identified in the meaningful loops of our network (**Figure 1.F and Figure S4),** i.e., BMI; genera *Allistipes* and *Collinsella*; aminoacids hydroxyproline, prolyhydroxyproline, aspartate; lipids Sphingomyelin (2)/(3) ; and SNPs rs34723569, 3:61213993/3:61219865, and rs6767126.
2. Randomly partition the subset of data in a training set, containing 70% of the samples, and a test set, containing the remaining 30%
3. Fit a linear model with one of the variables as the response, and the rest as predictors, adjusting for sex, race, and study site as covariates on the train set.
4. Using the trained model, generate predictions on the test set with complete measurements, and calculate the mean-squared error and $R^{2}$ with respect to the real measurements.
5. Repeat step 3 for each of the selected variables. When the response is a highly correlated with another variable of the same omic, remove the second one from the predictors. That is, when the predictor is hydroxyproline, prolyhydroxyproline will not be used as a predictor and viceversa. This is the case for aminoacids hydroxyproline, prolyhydroxyproline, lipids Sphingomyelin (2) and (3), and SNPS 3:61213993 and 3:61219865.

## List of abbreviations

body mass index (BMI)

microbiome-genome wide association studies (mGWAS)

Vitamin D Antenatal Asthma Reduction Trial (VDAART)

principal components (PCs)

Minor allele count (MAC)

microbiome composition PCs (MCPCs)

false discovery rate (FDR)

dipeptidyl peptidase (DPPIV)

Environmental influences on Child Health Outcomes (ECHO)

## References

[1] S. T. Weiss, Randomized Trial: Maternal Vitamin D Supplementation to Prevent Childhood Asthma (VDAART), Clinical trial registration No. NCT00920621, clinicaltrials.gov, 2023.

[2] A. A. Litonjua, N. E. Lange, V. J. Carey, S. Brown, N. Laranjo, G. T. O’Connor, et al., *The Vitamin D Antenatal Asthma Reduction Trial (VDAART): Rationale, Design, and Methods of a Randomized, Controlled Trial of Vitamin D Supplementation in Pregnancy for the Primary Prevention of Asthma and Allergies in Children*, Contemp Clin Trials **38**, 37 (2014).

[3] M. Vogel, *Childsds: Data and Methods Around Reference Values in Pediatrics*, (2022).

[4] Center for Diseases Control and Prevention, *BMI for Age-Weight Status Categories and the Corresponding Percentiles*, https://www.cdc.gov/healthyweight/assessing/bmi/childrens_bmi/about_childrens_bmi.html.

[5] E. A. Bridgewater BR, *High Resolution Mass Spectrometry Improves Data Quantity and Quality as Compared to Unit Mass Resolution Mass Spectrometry in High-Throughput Profiling Metabolomics*, Metabolomics **04**, (2014).

[6] K. Blighe, B. L. Chawes, R. S. Kelly, H. Mirzakhani, M. McGeachie, A. A. Litonjua, et al., *Vitamin D Prenatal Programming of Childhood Metabolomics Profiles at Age 3 y*, Am J Clin Nutr **106**, 1092 (2017).

[7] J. Xia, N. Psychogios, N. Young, and D. S. Wishart, *MetaboAnalyst: A Web Server for Metabolomic Data Analysis and Interpretation*, Nucleic Acids Res **37**, W652 (2009).

[8] B. Howie, C. Fuchsberger, M. Stephens, J. Marchini, and G. R. Abecasis, *Fast and Accurate Genotype Imputation in Genome-Wide Association Studies through Pre-Phasing*, Nat Genet **44**, 8 (2012).

[9] *Ancestry Estimation and Control of Population Stratification for Sequence-Based Association Studies | Nature Genetics*, https://www-nature-com.ezp-prod1.hul.harvard.edu/articles/ng.2924.

[10] C. Martino, J. T. Morton, C. A. Marotz, L. R. Thompson, A. Tripathi, R. Knight, et al., *A Novel Sparse Compositional Technique Reveals Microbial Perturbations*, MSystems **4**, e00016 (2019).

[11] S. Sanna, A. Kurilshikov, A. van der Graaf, J. Fu, and A. Zhernakova, *Challenges and Future Directions for Studying Effects of Host Genetics on the Gut Microbiome*, Nat Genet **54**, 2 (2022).

[12] P. J. McMurdie and S. Holmes, *Phyloseq: An R Package for Reproducible Interactive Analysis and Graphics of Microbiome Census Data*, PLOS ONE **8**, e61217 (2013).

[13] *Maaslin2*, http://bioconductor.org/packages/Maaslin2/.

[14] *Igraph – Network Analysis Software*, https://igraph.org/.
